# UHPLC-MS/MS method for pharmacokinetic and bioavailability determination of five bioactive components in raw and various processed products of *Polygala tenuifolia* in rat plasma

**DOI:** 10.1080/13880209.2020.1818790

**Published:** 2020-09-21

**Authors:** Xin Zhao, Baoxin Xu, Peng Wu, Pan Zhao, Changchuan Guo, Yueli Cui, Yanxue Zhang, Xuelan Zhang, Huifen Li

**Affiliations:** a School of Chinese Pharmacy, Shandong University of Traditional Chinese Medicine, Jinan, China; b Shandong Institute for Food and Drug Control, Jinan, China; c Shandong Provincial Collaborative Innovation Center for Quality Control and Construction of the Whole Industrial Chain of Traditional Chinese Medicine, Jinan, China

**Keywords:** Liquorice boiling, honey stir-baking, sibiricose A5, sibiricose A6, 3,6′-disinapoyl sucrose, tenuifoliside A, 3,4,5-trimethoxycinnamic acid

## Abstract

**Context:**

Sibiricose A5 (A5), sibiricose A6 (A6), 3,6′-disinapoyl sucrose (DSS), tenuifoliside A (TFSA) and 3,4,5-trimethoxycinnamic acid (TMCA) are the main active components of *Polygala tenuifolia* Willd. (Polygalaceae) (PT) that are active against Alzheimer's disease.

**Objective:**

To compare the pharmacokinetics and bioavailability of five active components in the roots of raw PT (RPT), liquorice-boiled PT (LPT) and honey-stir-baked PT (HPT).

**Materials and methods:**

The median lethal dose (LD_50_) was evaluated through acute toxicity test. The pharmacokinetics of five components after oral administration of extracts of RPT, LPT, HPT (all equivalent to 1.9 g/kg of RPT extract for one dose) and 0.5% CMC-Na solution (control group) were investigated, respectively, in Sprague-Dawley rats (four groups, *n* = 6) using UHPLC-MS/MS. In addition, the absolute bioavailability of A5, A6, DSS, TFSA and TMCA after oral administration (7.40, 11.60, 16.00, 50.00 and 3.11 mg/kg, respectively) and intravenous injection (1/10 of the corresponding oral dose) in rats (*n* = 6) was studied.

**Results:**

The LD_50_ of RPT, LPT and HPT was 7.79, 14.55 and 15.99 g/kg, respectively. *AUC*_0-_*_t_* of RPT, LPT and HPT were as follows: A5 (433.18 ± 65.48, 680.40 ± 89.21, 552.02 ± 31.10 ng h/mL), A6 (314.55 ± 62.73, 545.76 ± 123.16, 570.06 ± 178.93 ng h/mL) and DSS (100.30 ± 62.44, 232.00 ± 66.08, 197.58 ± 57.37 ng h/mL). The absolute bioavailability of A5, A6, DSS, TFSA and TMCA was 3.25, 2.95, 2.36, 1.17 and 42.91%, respectively.

**Discussion and conclusions:**

The pharmacokinetic and bioavailability parameters of each compound can facilitate future clinical studies.

## Introduction

The root of *Polygala tenuifolia* Willd. (Polygalaceae) (PT) is a common traditional Chinese medicine that has been listed as a nootropic, anti-inflammatory and anti-psychotic medicine to cure insomnia (Yao et al. 2010), forgetfulness, neurasthenia, coughing and soreness caused by heart and kidney disharmony (Li et al. 2008; Leem and Oh 2015). Raw PT (RPT) presents the side effects of gastrointestinal toxicity and throat irritation, and it requires processing before being prescribed in Chinese clinical practices. In modern times, the most common processing methods of PT include heartwood discarding, liquorice boiling and honey stir-baking. Among them, liquorice-boiled PT (LPT) and honey-stir-baked PT (HPT) are commonly used in clinics. RPT and LPT are listed in the Chinese Pharmacopoeia (Chinese Pharmacopoeia Commission 2015), and HPT is listed in the National Regulations for the Processing of Traditional Chinese Medicine (Chinese Drug Administration 1988). According to the theories of traditional Chinese medicine, LPT can reduce the side effects of RPT on pharyngeal irritation and gastrointestinal stimulation and promote peace of mind and intellectual development. LPT is widely used to treat neurological disorders, such as palpitations, restlessness and trances. Additionally, HPT can eliminate adverse reactions and enhance the effect of resolving phlegm and relieving cough. It is mainly used to treat chronic bronchitis, coughs, phlegm and other diseases. Processing can reduce the gastrointestinal motility disorder of RPT and enhance its neuroprotective effect (Yang et al. 2011).

Recent pharmacological studies have suggested that PT and its extracted fractions have significant effects regarding neuroprotection (Wang et al. 2019), anti-amnesia (Zhang et al. 2008), anti-depressant properties (Cao et al. 2018) and relieving the symptoms of extreme fear in rats (Shin et al. 2018). The main chemical components in PT include triterpenoid saponins, oligosaccharide esters and organic acids, of which oligosaccharide esters and organic acids are beneficial for the central nervous system (Liu et al. 2015; Chen et al. 2016). Sibiricose A5 (A5), sibiricose A6 (A6), 3,6′-disinapoyl sucrose (DSS) and tenuifoliside A (TFSA) are representative oligosaccharide esters in PT, while 3,4,5-trimethoxycinnamic acid (TMCA) is a representative organic acid, which showed anti-seizure (Chen et al. 2016), anti-stroke (Li et al. 2017), anti-inflammatory (Kim et al. 2013), neuroprotective (Liu et al. 2015) and anti-depressant-like effects (Hu et al. 2010). To date, several pharmacokinetic studies about the active components of PT have been reported. For instance, it has been reported that the pharmacokinetics and bioavailability of three *Polygala* saponin hydrolysates were determined by a sensitive LC-MS/MS method (Wang et al. 2015). Moreover, the pharmacokinetics of TFSA, tenuifoliside C, polygalaxanthone III and tenuifolin from PT extract in rat plasma were acquired using LC-MS/MS (Lin et al. 2014b). Recently, our research group established a UHPLC-MS/MS method for the simultaneous and quantitative determination of A5, A6, DSS, TFSA, tenuifoliside B, tenuifoliside C and TMCA in rat plasma (Xu et al. 2018b). However, pharmacokinetic comparisons of the main active components among raw and various processed products of PT have not been reported. Additionally, the bioavailability studies involving oligosaccharide esters and organic acids of PT are rare.

In this study, a highly specific, sensitive and rapid UHPLC-MS/MS method was successfully applied to the pharmacokinetic comparisons of A5, A6, DSS, TFSA and TMCA in rat plasma after oral administration of extracts from RPT, LPT and HPT for the first time, as well as the absolute bioavailability of the five compounds in rat plasma after oral and intravenous (i.v.) administration. It was expected that the study would provide a scientific basis for the processing mechanism of PT and guide reasonable clinical applications of processed PT.

## Materials and methods

### Chemicals and reagents

A5 and A6 were supplied by Chengdu Biopurify Phytochemicals Co., Ltd. (Chengdu, China). The reference standard of TFSA was supplied by Shanghai Yilin Biological Technology Co., Ltd. (Shanghai, China). DSS and TMCA were obtained from Chengdu Chroma Biotechnology Co., Ltd. (Chengdu, China). The internal standard (IS, salicylic acid) was provided by Shanghai Yuanye Biological Technology Co., Ltd. (Shanghai, China). The purity of each reference standard was higher than 98%. HPLC-grade water was obtained using a Milli-Q water purification system (Millipore, Billerica, MA). In addition, other reagents, such as acetonitrile, methanol and ammonium acetate, were all of HPLC grade and were supplied by Merck Co., Ltd. (Darmstadt, Germany).

### Animals

Adult Sprague-Dawley (SD) rats weighing 180–220 g were obtained from Pengyue Experimental Animal Centre (Jinan, China). The study protocol was approved by the Committee on Animal Care and Usage of Shandong University of Traditional Chinese Medicine (ethical committee approval numbers: SDUTCM20190304003, approval date (4 March 2019) and SDUTCM20190409001, approval date (9 April 2019)). Animals were kept in an environmentally controlled breeding room (temperature: 25 ± 2 °C, humidity: 60 ± 10%, 12 h dark-light cycle) for 7 consecutive days. Despite fasting overnight prior to administration, water was provided *ad libitum* throughout the experiment.

### Sample collection

All RPT samples (five batches of samples) were purchased from Shandong Baiweitang Chinese Medicine Pieces Co., Ltd. in October 2018 (Jinan, China) and were authenticated by Li Feng, a professor at Shandong University of Traditional Chinese Medicine (Jinan, China). Five batches of samples were collected from Shandong and Shanxi provinces. Five voucher specimens (Nos. SDCM-YZ2018121101, SDCM-YZ2018121102, SDCM-YZ2018121103, SDCM-YZ2018121104 and SDCM-YZ2018121105) were deposited at the Herbarium of Traditional Chinese Medicine, School of Pharmacy, Shandong University of Traditional Chinese Medicine. Each batch of samples was divided into three groups: one group was RPT, and the other two groups were used for the preparation of LPT and HPT.

LPT and HPT were prepared in the laboratory. The processing procedure of LPT samples was as follows: the liquorice was boiled twice in water for 0.5 h each time. Then, the two filtrates were combined and concentrated to 0.1 g of liquorice per 1 mL of decoction. Subsequently, 150 g of PT was moistened for 1 h with 90 mL of liquorice decoction and 500 mL of water and subsequently boiled for 5 h until it absorbed the liquid decoction completely. Then, LPT samples were dried under vacuum at 50 °C. The HPT samples were obtained as follows: first, 150 g of PT was mixed with 30 g of refined honey and a moderate amount of water and then moistened for 1 h and stir-heated until the PT was dark yellow.

### Preparation of sample extracts

Raw and various processed products of PT were smashed into powder (40 meshes). One hundred grams of powder from each sample was accurately weighed and extracted by reflux three times with 50% ethanol of 10, 8 and 8 times the volume of the samples, each time for 0.5 h. The combined filtrate was concentrated and dried in vacuum at 50 °C. The five components in the raw and various processed products of PT extracts were quantitatively determined by HPLC as previously established by our team (Meng et al. 2015); the chromatogram is illustrated in Supplementary Figure S1.

### Acute toxicity study of extracts from raw and various processed products of PT

A total of 160 SD rats were randomly divided into 16 groups (half male and half female, *n* = 10). All oral administration samples were mixed with 0.5% CMC-Na (w/v). The test groups were subjected to one-time intragastric administration of different doses of raw and various processed products of PT extracts at the following dose ranges: RPT was from 4.15 to 13.00 g/kg body weight, LPT and HPT were from 7.99 to 25.00 g/kg body weight. Control rats were treated with the vehicle alone (volume: 10 mL/kg). Then, the rats were observed for 14 days to record the mortality, body weight changes, and signs of toxicity. The median lethal dose (LD_50_), 95% confidence interval (CL) and probit-log (dose) equations were calculated by SPSS analysis system (version 23.0, IBM Co., Ltd. Armonk, NY).

### Chromatographic and UHPLC-MS/MS conditions

A Thermo Hypersil Gold C18 column (3.0 × 100 mm, i.d.; 3 μm) was used for chromatographic separation. An aqueous 10 mM ammonium acetate solution (A) and acetonitrile (B) were selected as the mobile phase components. The optimal gradient program involved the following steps: 5% B (0–1 min), changing to 85% B (1–3.5 min), 85% B (3.5–4.0 min), changing to 5% B (4.0–4.1 min) and 5% B (4.1–5.0 min). The flow rate was set to 0.4 mL/min, the autosampler was maintained at 4 °C and the injection volume was 5 μL.

**Figure 1.**
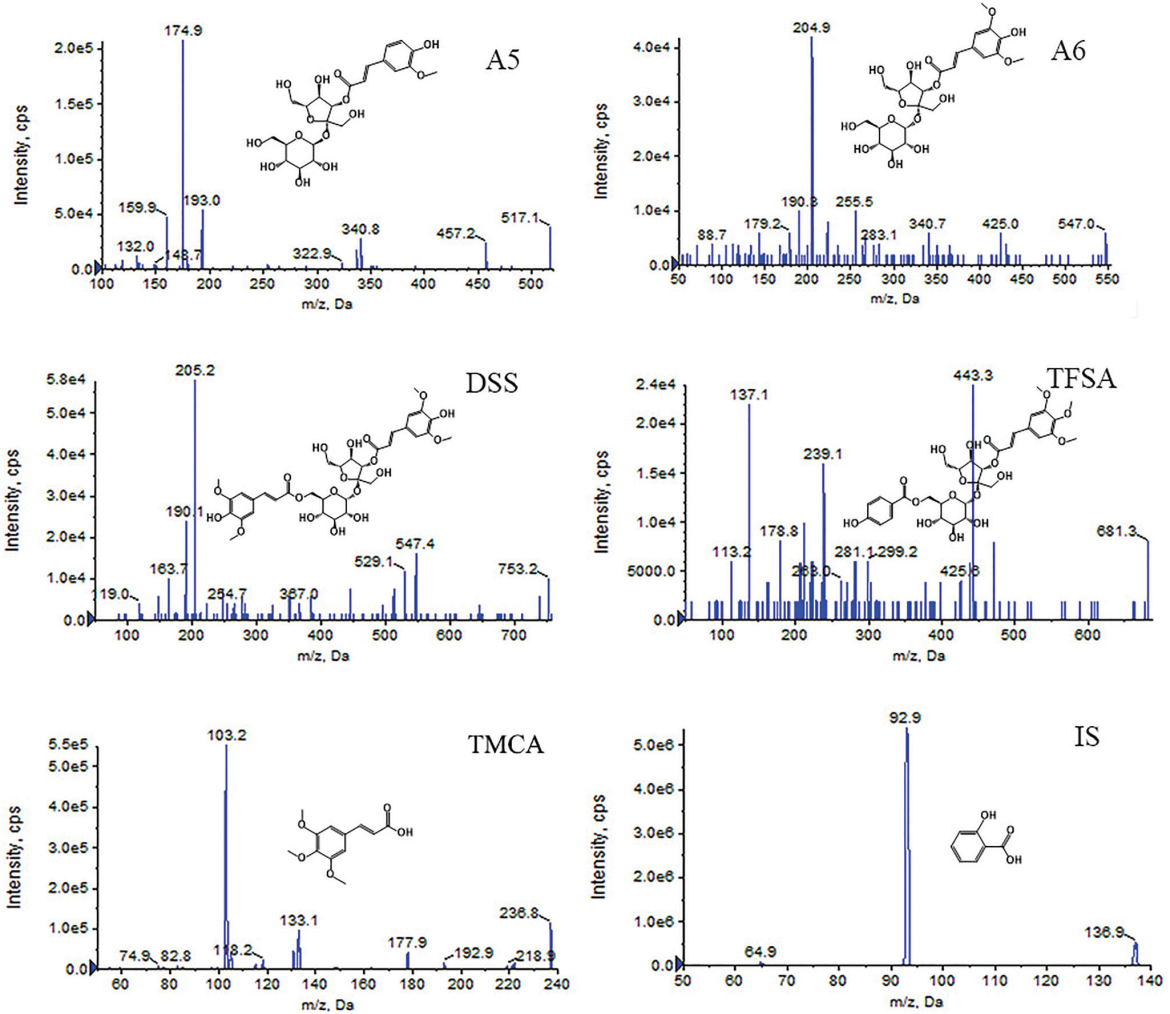
Chemical structures and positive product ion mass spectra of the five analytes and IS.

UHPLC-MS/MS was conducted by an Applied Biosystems (AB) Sciex Exion LC system (Foster City, CA) connected to a 6500 + triple quad tandem mass spectrometer with an electrospray ionization source. All detection and quantifications were carried out in positive multiple reaction monitoring (MRM) mode. The optimized parameters were programmed as follows: ion source temperature, 500 °C; curtain gas pressure, 30 kPa; ion source gas 1 pressure, 55 kPa; ion source gas 2 pressure, 55 kPa; and ion spray voltage, −3500 V. The chemical structures and ion mass spectra of targeted compounds are shown in Figure 1. The precursor ion and product ion were *m/z* 517.1 → 174.9 for A5, *m/z* 547.0 → 204.9 for A6, *m/z* 753.2 → 205.2 for DSS, *m/z* 681.3 → 443.3 for TFSA, *m/z* 236.8 → 103.2 for TMCA and *m/z* 136.9 → 92.9 for IS. All data were acquired by using AB Sciex Analyst software (version 1.6.3).

### Preparation of standard solutions, calibration solutions and quality control samples

Stock standards of A5, A6, DSS, TFSA, TMCA and IS were prepared in methanol at a concentration of 1 mg/mL. A series of working standard solutions of the targeted compounds ranging from 10 to 20,000 ng/mL and the IS solution at 600 ng/mL were prepared by diluting the stock solutions with methanol and maintained at −20 °C. Calibration standards were freshly prepared by spiking the series of standard solutions (30 μL) including the IS into blank rat plasma (270 μL) to achieve concentrations of 1, 10, 50, 100, 500, 1000, 1600 and 2000 ng/mL. Quality control (QC) samples were prepared independently at low (2 ng/mL), medium (600 ng/mL) and high (1600 ng/mL) concentrations following the same method for the five compounds.

### Plasma sample preparation

After thawing the frozen blood samples at room temperature, a 100 μL aliquot of plasma was spiked with 50 μL of the IS working solution in 2 mL EP tubes, and 750 μL of methanol was added into each of the tubes. Then, these tubes were mixed on a multi-tube Vortexer for 0.5 min before centrifugation at 11,800×*g* for 10 min. Finally, 200 μL of the obtained supernatant was transferred to a fresh autosampler vial, and then 5 μL of the sample was injected for analysis.

### Method validation

The UHPLC-MS/MS method validation was performed in accordance with an internal standard operation procedure based on the US Food and Drug Administration (FDA) guidelines (US Food and Drug Administration 2013). The validation parameters, including the selectivity, linearity, lowest limit of quantification (LLOQ), accuracy, precision, extraction recovery, matrix effect and storage stability, were measured accordingly.

### Pharmacokinetic study

#### Pharmacokinetic study on five components of extracts from raw and various processed products of PT

Male SD rats were randomly divided into four groups (*n* = 6 in each group), including the RPT group, LPT group, HPT group and control group. All oral administration samples were mixed with 0.5% CMC-Na (w/v) and then given to rats at a dose equivalent to 1.9 g/kg of RPT extract. The control group was orally treatment with equal volume of 0.5% CMC-Na solution. After a single administration, all blood samples were collected from the orbital vein into heparinized centrifuge tubes before administration (0 h) and at 0.083, 0.17, 0.25, 0.5, 0.75, 1, 1.5, 2, 3, 5, 8 and 12 h after administration and then centrifuged at 4 °C and 11,800×*g* for 10 min. The separated supernatant samples were preserved at −80 °C until they were measured.

#### Bioavailability of five compounds of PT in rat plasma

The SD rats were randomly divided into oral and i.v. groups for A5, A6, DSS, TFSA and TMCA administration (*n* = 6 in each group). The oral doses of A5, A6, DSS, TFSA and TMCA groups were 7.40, 11.60, 16.00, 50.00 and 3.11 mg/kg, respectively, and the corresponding i.v. doses were 0.74, 1.16, 1.60, 5.00 and 0.31 mg/kg, respectively. Blood samples were collected from the suborbital vein into heparinized tubes before and 0, 0.083, 0.17, 0.25, 0.5, 1, 2, 3, 5 and 8 h after administration. The treatment method of blood samples was the same as that mentioned above.

#### Pharmacokinetic data analysis

The elementary pharmacokinetic parameters of plasma samples were analyzed by DAS 2.0 pharmacokinetic software. One-way analysis of variance (ANOVA) was used for statistical comparison for pharmacokinetic and interaction studies. A *p*-value < 0.05 indicated a significant difference. The main parameters considered were as follows: *AUC*_0_*_–∞_* (the area under the blood concentration-time curve from zero to infinity initial plasma concentration), *MRT* (the mean residence time), *C*_max_ (peak plasma concentration) and *T*_max_ (time to reach *C*_max_ for oral dose), *t*_1/2_ (elimination half-life) (He et al. 2015). The absolute bioavailability (*F*) was evaluated as follows:

$$F\left(\%\right)=\left({AUC}_{\text{oral}}\times {\text{Dose}}_{i.v.}\right)/\left({AUC}_{i.v.}\times {\text{Dose}}_{\text{oral}}\right)\times 100\%$$

## Results

### Determination of sample content

The contents of five components in the raw and various processed products of PT extracts are summarized in Table 1. Compared with the RPT, the average contents of A5, A6, DSS and TFSA in the various processed products of PT decreased to different degrees, while the average content of TMCA in LPT was significantly increased, and the average content of TMCA in HPT was almost unchanged.

**Table 1.** Contents of the five components in the raw and various processed products of PT extracts (mg/g; mean ± SD, *n* = 5).

Analytes	RPT	LPT	HPT
A5	1.71 ± 0.011	1.27 ± 0.009	1.43 ± 0.010
A6	0.94 ± 0.006	0.89 ± 0.008	0.87 ± 0.009
DSS	4.37 ± 0.017	3.37 ± 0.014	4.28 ± 0.015
TFSA	2.07 ± 0.013	1.89 ± 0.010	1.99 ± 0.011
TMCA	0.07 ± 0.003	0.58 ± 0.004	0.07 ± 0.006

### Acute toxicity of oral extracts from raw and various processed products of PT

Acute toxicity test was conducted to preliminarily evaluate the safety of extracts from raw and various processed products of PT. The death in the acute toxicity test is reported in Table 2. There was no obvious intoxication reaction when rats were treated with extracts from raw and various processed products of PT at low dose. However, oral administration of high dose oral administration samples led to different toxic symptoms in rats, such as flatulence, gastric enlargement, thinning and necrosis of intestinal wall, edoema and haemolysis in the second half of small intestine and other symptoms. In addition, compared with the negative control group, the body weight of each dose group reduced. On the 15th day after administration, the morphology of other major organs in rats showed no obvious abnormality. Based on the Bliss method, the LD_50_ of RPT, LPT and HPT was 7.79 g/kg (CL: 5.11–9.38 g/kg), 14.55 g/kg (CL: 8.03–18.21 g/kg) and 15.99 g/kg (CL: 10.74–20.23 g/kg), respectively. The LD_50_ regression equation of RPT, LPT and HPT for oral administration was *y* = −7.695 + 8.632 × log (*d*), *y* = −8.904 + 7.658 × log (*d*) and *y* = −8.035 + 6.675 × log (*d*), respectively.

**Table 2.** The death by a single oral administration of raw and various processed products of PT extracts on SD rats in acute toxicity test (*n* = 10).

Group	Dose (g/kg)	Death	LD_50_ (g/kg)	95% CL (g/kg)
Control	0	0/10	0	0
RPT	13.00	10/10	7.79	5.11–9.38
	9.77	7/10		
	7.35	5/10		
	5.53	1/10		
	4.15	0/10		
LPT	25.00	10/10	14.55	8.03–18.21
	18.80	7/10		
	14.13	5/10		
	10.63	2/10		
	7.99	0/10		
HPT	25.00	9/10	15.99	10.74–20.23
	18.80	6/10		
	14.13	5/10		
	10.63	1/10		
	7.99	0/10		

### Methods validation result

#### Selectivity

**Figure 2.**
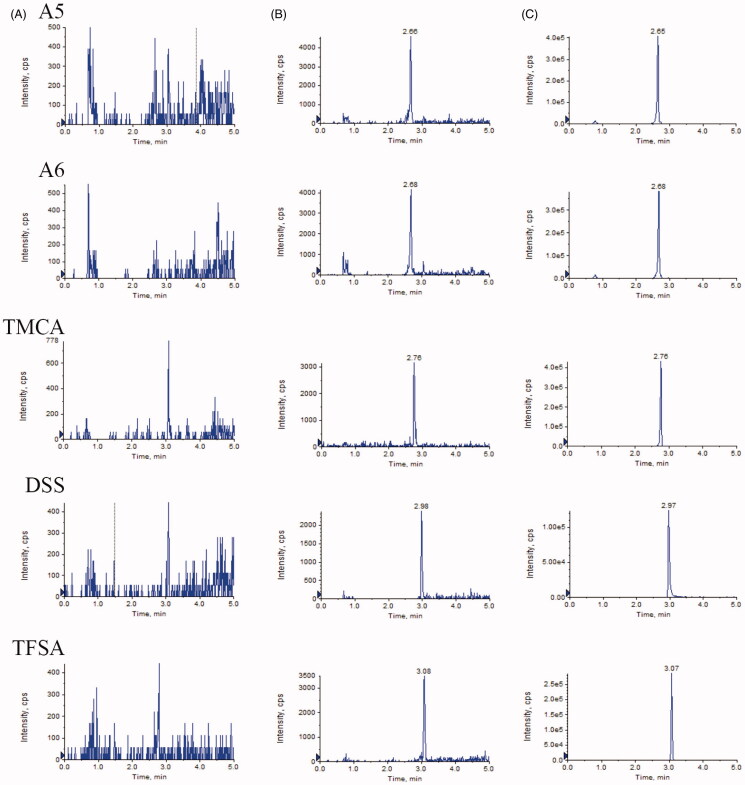
Representative MRM chromatograms of the five analytes in rat plasma: blank plasma (A); blank samples spiked with the five analytes at LLOQs (B); plasma sample obtained 15 min after oral administration (C).

Representative chromatograms of blank plasma, blank plasma spiked with five reference compounds at the LLOQs, and rat plasma obtained 15 min after oral administration are displayed in Figure 2. Under the optimized conditions, A5, A6, TMCA, DSS and TFSA eluted at 2.65, 2.68, 2.76, 2.97 and 3.07 min, respectively. No interference from endogenous substances in the plasma was observed at the retention time of each analyte.

### Linearity and lower limit of quantification

Linearity was expressed by plotting the peak-area ratio of each compound/IS versus the concentration (*y* was used to express the peak-area ratio of each analyte to that of the IS, and *x* was used to represent the plasma concentration of each analyte). The calibration curves were fitted by a weighed least squares linear regression (1/*x*^2^) method. The method possessed good linearity for the five compounds over concentrations ranging from 1 to 2000 ng/mL with a high correlation coefficient (*r* > 0.994). All acquired values are described in Supplementary Table S1. The LLOQs for the five compounds were lower than 1 ng/mL, which would contribute to obtaining accurate pharmacokinetic parameters in rat plasma.

### Accuracy and precision

The results on a single day (intra-day) and three consecutive days (inter-day) were utilised to determine the precision and accuracy analysis of three concentration levels of QC samples, which are listed in Supplementary Table S2. The accuracy and precision were calculated as the relative error (RE, %) and relative standard deviation (RSD, %), respectively. All data indicated that the intra- and inter-day accuracies were within acceptable limits of −4.6 to 5.3% and −7.1 to 6.8%, respectively, at all tested concentrations, and the corresponding precisions ranged from 2.5 to 6.9% and 0.9 to 7.4%, respectively. All the obtained values were within acceptable criteria (±15%), indicating that the established method had high accuracy and precision.

### Extraction recovery and matrix effect

The extraction recovery was measured by comparing the peak area of each analyte in pre-extraction samples with those in post-extraction spiked samples. The matrix effect was evaluated by comparing the peak area of each analyte in spiked plasma samples against those of the corresponding reference standard solutions. The extraction recovery and matrix effect of each analyte were determined at three QC levels with six replicates. The relevant data are summarized in Supplementary Table S3. The extraction recoveries of the five analytes were within the range of 85.2–103.4%, and the RSDs were no more than 6.9%, while the matrix effects were within 85.5–103.3%, and the RSDs were less than 7.2%, which indicated that methanol was an ideal precipitation agent and that there was no significant matrix effect in the analytical method.

### Stability

Stability studies included short-term stability (5 h at room temperature, i.e. 25 °C), freeze-thaw stability (three consecutive freeze-thaw cycles from −20 °C to room temperature), autosampler stability (24 h storage in the autosampler at 4 °C) and long-term stability (4 weeks storage at −80 °C). The stability data of all analytes are presented in Supplementary Table S4. The obtained results demonstrated that the developed method had good stability for routine analysis.

### Application to a pharmacokinetic analysis

**Figure 3.**
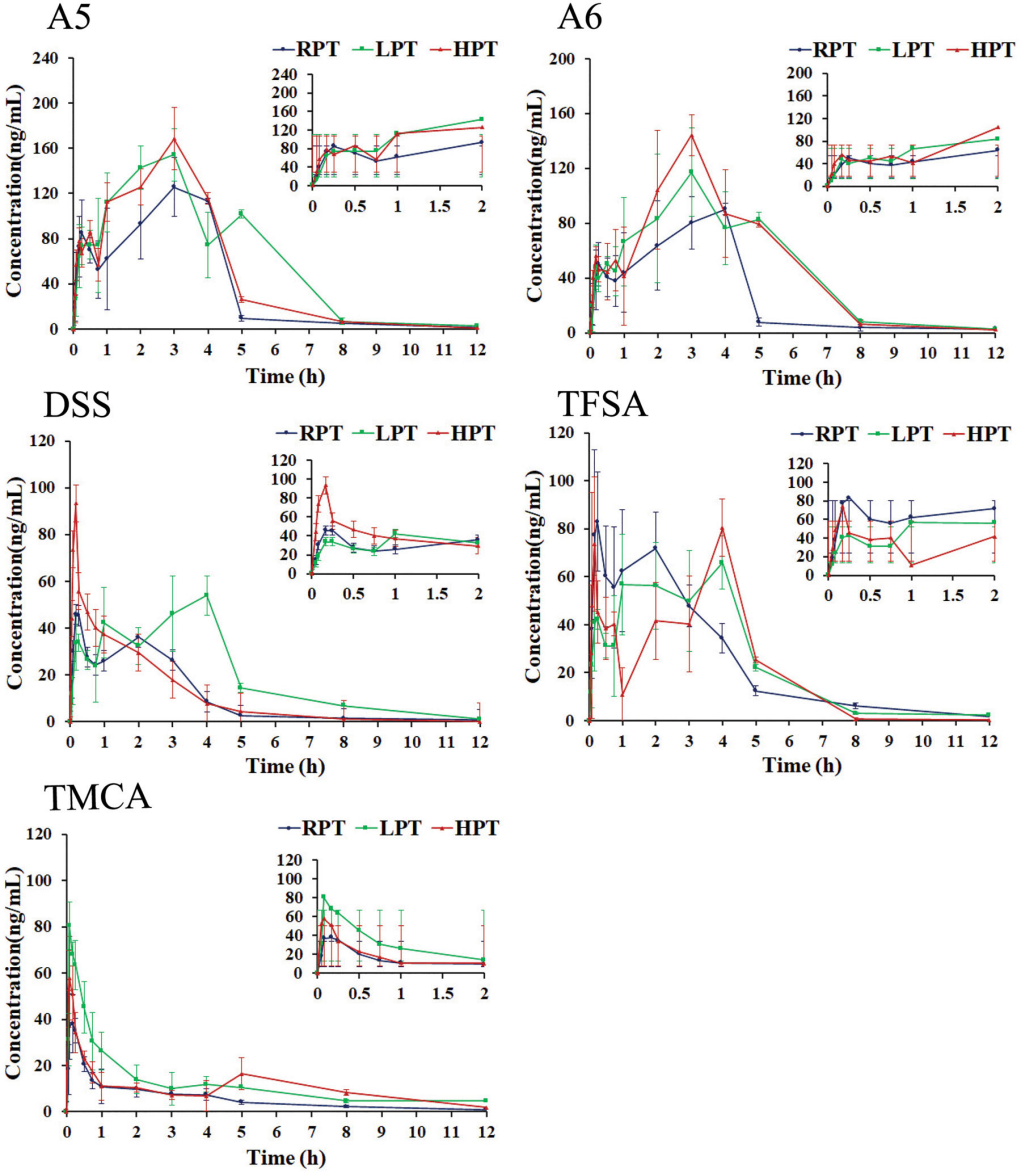
Mean concentration-time profiles of five analytes in rat plasma after oral administration of the extracts from raw and processed products of PT. Insets describe initial 2 h profiles for the five analytes. Each point represents the mean ± SD of six determinations.

The UHPLC-MS/MS method was sensitive enough to analyze A5, A6, DSS, TFSA and TMCA in rat plasma after a single administration. The mean concentration-time profiles of five components in rat plasma after oral administration of extracts from raw and various processed products of PT are described in Figure 3, and the pharmacokinetic parameters are displayed in Table 3.

**Table 3.** Pharmacokinetic parameters of the five components in rat plasma after oral administration of extracts from raw and various processed products of PT (mean ± SD, *n* = 6).

Group	Analytes	*AUC*_0-_*_t_* (ng h/mL)	*AUC*_0-∞_ (ng h/mL)	*MRT*_0-_*_t_* (h)	*t*_1/2_ (h)	*T*_max_ (h)	*C*_max_ (ng/mL)
RPT	A5	433.18 ± 65.48	437.13 ± 71.54	2.63 ± 0.14	1.49 ± 0.26	2.60 ± 0.71	191.10 ± 109.10
	A6	314.55 ± 62.73	325.06 ± 56.16	2.83 ± 0.48	1.86 ± 0.97	2.80 ± 0.45	94.58 ± 26.95
	DSS	100.30 ± 62.44	102.48 ± 60.78	2.45 ± 0.79	2.63 ± 1.07	0.80 ± 0.89	74.78 ± 46.60
	TFSA	289.45 ± 134.21	298.77 ± 142.23	2.83 ± 0.38	5.26 ± 2.20	0.72 ± 0.61	132.08 ± 88.42
	TMCA	91.66 ± 29.15	131.86 ± 33.41	4.37 ± 0.53	6.93 ± 2.96	0.13 ± 0.05	38.75 ± 13.78
LPT	A5	680.40 ± 89.21^a^	689.70 ± 81.13^a^	3.32 ± 0.05^a^	2.20 ± 0.31^a^	2.00 ± 0.61	173.2 ± 28.50
	A6	545.76 ± 123.16^a^	559.88 ± 131.61^a^	3.61 ± 0.57^a^	2.67 ± 0.93	2.65 ± 1.73	135.92 ± 28.06^a^
	DSS	232.00 ± 66.08^a^	233.88 ± 67.24^a^	3.13 ± 1.04	1.53 ± 0.34	2.30 ± 2.04	74.08 ± 30.21
	TFSA	284.15 ± 184.89	297.18 ± 193.52	2.92 ± 0.58	4.92 ± 3.48	1.56 ± 1.20	107.15 ± 47.67
	TMCA	134.65 ± 33.35	172.85 ± 40.39	3.56 ± 0.25^a^	6.82 ± 1.82	0.10 ± 0.04	80.65 ± 11.07^a^
HPT	A5	552.02 ± 31.10^a^	553.32 ± 47.08^a^	2.64 ± 0.18	1.23 ± 0.21	2.00 ± 0.00^a^	168.52 ± 16.15
	A6	570.06 ± 178.93^a^	572.06 ± 178.99^a^	3.21 ± 0.78	1.43 ± 0.12	3.20 ± 1.64	183.48 ± 30.78^a^
	DSS	197.58 ± 57.37^a^	198.34 ± 59.21^a^	1.54 ± 0.29^a^	1.23 ± 0.30^a^	0.17 ± 0.00	116.19 ± 14.49
	TFSA	279.46 ± 120.03	281.26 ± 126.1	3.06 ± 0.57	1.28 ± 0.86	3.29 ± 1.13	98.41 ± 38.24
	TMCA	123.95 ± 41.30	183.54 ± 137.31	4.19 ± 0.91	3.73 ± 2.34	0.07 ± 0.02	63.17 ± 14.24^a^

^a^Indicated *p* < 0.05 as compared with the RPT group.

Double peaks were observed from the curves of A5, A6 and TFSA in the RPT, LPT, and HPT groups, and additional peaks were detected from the curves of DSS in the RPT, and LPT groups (see Figure 3). Compared with the RPT group, the *AUC*_0_*_-t_* values of A5, A6 and DSS, along with the *C*_max_ value of A6, were obviously elevated (*p* < 0.05) in the LPT group and HPT group (Table 3). In addition, compared with the RPT group, the *AUC*_0_*_-t_* and *C*_max_ values of TFSA were also decreased, while the *C*_max_ values of TMCA were remarkably increased (*p* < 0.05) in the other two processed products of PT groups. Moreover, the *MRT* values of A5 and A6, along with the *t*_1/2_ value of A5, were still remarkably improved (*p* < 0.05) in the LPT group compared to those of the RPT group. Additionally, the *MRT* and *t*_1/2_ values of DSS were significantly elevated (*p* < 0.05) in the HPT group compared with those in the RPT group.

**Figure 4.**
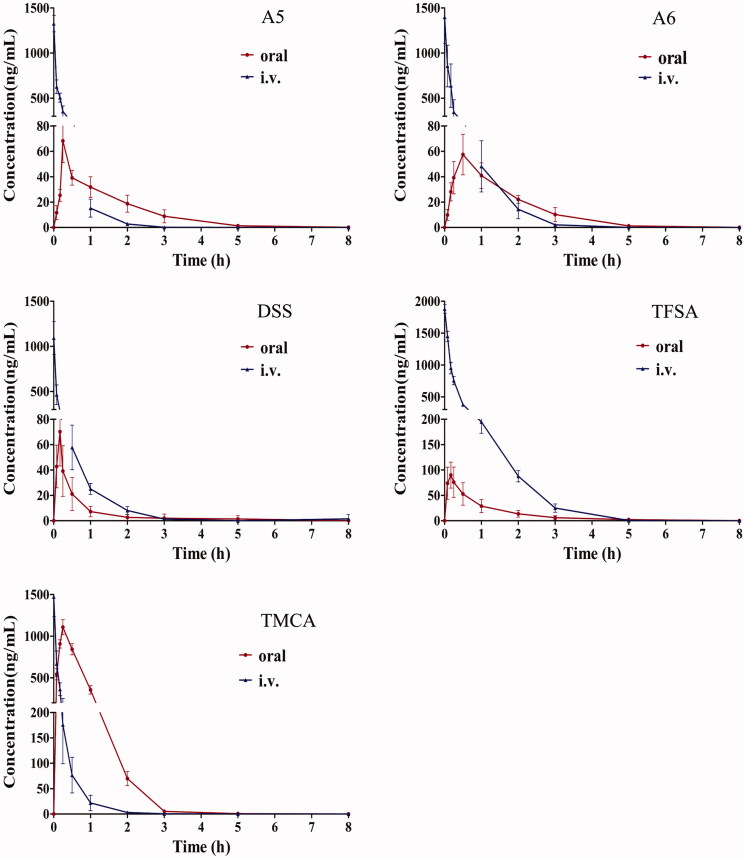
Mean concentration–time profiles of five compounds in rat plasma after oral and intravenous administration. Each point represents the mean ± SD of six determinations.

The mean concentration–time profiles of five compounds following oral and i.v. administration are depicted in Figure 4, and the pharmacokinetic parameters are summarized in Table 4. The absolute bioavailability of A5, A6, DSS, TFSA and TMCA was 3.25, 2.95, 2.36, 1.17 and 42.91%, respectively. The *t*_1/2_ values of A5 were prolonged compared with those of A6 after oral and i.v. administration, which indicated that the elimination of A5 was slower than that of A6. Moreover, A5 showed a higher *AUC*^Δ^_0_*_-t_* (^Δ^ indicated dose-normalized value) and *C*^Δ^_max_ than A6. Comparing the pharmacokinetic parameters of DSS with those of A6, we found that the *AUC*^Δ^_0_*_-t_* values of A6 were approximately three times larger than those of DSS after both routes of administration, with a higher absolute bioavailability of 2.95%, despite its relatively lower absorbed ratio. Notably, the TFSA was rapidly absorbed and reached its peak plasma concentration approximately 0.19 h after oral administration, with a bioavailability of 1.17%. However, the *AUC*^Δ^_0_*_-t_* and *C*^Δ^_max_ values of TMCA were much larger than those of TFSA after oral and i.v. administration, with a higher absolute bioavailability of 42.91%.

**Table 4.** Pharmacokinetic parameters of the five compounds after oral and intravenous administration in rat plasma (mean ± SD, *n* = 6).

	Oral administration					I.v. administration				
	A5	A6	DSS	TFSA	TMCA	A5	A6	DSS	TFSA	TMCA
Dose (mg/kg)	7.40	11.60	16.00	50.00	3.11	0.74	1.16	1.60	5.00	0.31
*AUC*_0-_*_t _*(ng h/mL)	88.71 ± 14.84	102.28 ± 27.79	40.17 ± 17.12	96.18 ± 42.39	968.46 ± 58.41	272.56 ± 42.30	347.01 ± 116.27	170.11 ± 19.35	818.97 ± 59.15	224.91 ± 60.89
*AUC*_0-∞_ (ng h/mL)	88.85 ± 14.82	102.34 ± 27.86	45.80 ± 21.31	96.19 ± 42.39	971.31 ± 59.80	272.57 ± 42.31	347.02 ± 116.27	171.20 ± 19.34	818.99 ± 59.17	226.50 ± 61.21
*MRT*_0-_*_t _* (h)	1.50 ± 0.19	1.44 ± 0.08	1.10 ± 0.91	1.20 ± 0.13	0.68 ± 0.03	0.25 ± 0.02	0.36 ± 0.09	0.46 ± 0.21	0.69 ± 0.05	0.25 ± 0.06
*t*_1/2_ (h)	1.27 ± 0.55	0.62 ± 0.16	1.01 ± 0.45	0.92 ± 0.21	0.65 ± 0.02	1.22 ± 0.07	0.59 ± 0.10	0.98 ± 0.27	0.49 ± 0.06	0.62 ± 0.13
*T*_max_ (h)	0.25 ± 0.00	0.50 ± 0.00	0.17 ± 0.00	0.19 ± 0.04	0.25 ± 0.00	－	－	－	－	－
*C*_max_ (ng/mL)	68.18 ± 17.04	57.46 ± 19.00	70.16 ± 27.02	93.96 ± 38.13	1108.20 ± 90.51	1320.40 ± 100.93	1397.40 ± 289.61	1092.60 ± 183.47	1877.80 ± 68.24	1472.60 ± 238.18
*AUC*^Δ^_0-_*_t_* (ng h/mL)^a^	11.99 ± 2.01	8.82 ± 2.40	2.51 ± 1.07	1.92 ± 0.85	311.40 ± 18.78	368.32 ± 57.16	299.15 ± 100.23	106.32 ± 12.09	163.79 ± 11.83	725.52 ± 196.42
*AUC*^Δ^_0-∞_ (ng h/mL)^a^	12.01 ± 2.00	8.82 ± 2.40	2.86 ± 1.33	1.92 ± 0.85	312.32 ± 19.23	368.34 ± 57.18	299.16 ± 100.23	107.00 ± 12.09	163.80 ± 11.83	730.65 ± 197.45
*C*^Δ^_max_ (ng/mL)^a^	9.21 ± 2.30	4.95 ± 1.64	4.39 ± 1.69	1.88 ± 0.76	356.33 ± 29.10	1784.32 ± 136.39	1204.66 ± 249.66	682.88 ± 120.92	375.56 ± 13.65	4750.32 ± 768.32
*F* (% of dose)	3.25	2.95	2.36	1.17	42.91					

^Δ^Indicated dose-normalized value.

## Discussion

In our preliminary experiments, the oral lethal potency of extracts from raw and various processed products of PT was RPT > LPT > HPT and their main toxic target organs were the stomach and small intestine. The acute toxicity test results indicated that processing technology could reduce the toxicity of RPT and improve its safety. Meanwhile, the results showed that the doses given to rats in the pharmacokinetic study are safe.

The double peak phenomenon of A5, A6, DSS and TFSA has been reported in previous studies (Lin et al. 2014a, 2014b; Meng et al. 2015). It is speculated that this may be ascribed to presystemic metabolism, gastrointestinal ‘absorption' window, enterohepatic circulation, gastric emptying changes and drug-drug interactions (Zhou 2003; Zhang et al. 2007). In addition, our team reported that DSS could be hydrolyzed to the secondary glycoside A6, and TFSA could be hydrolyzed to the small organic acid TMCA under processing conditions (Qu et al. 2018; Xu et al. 2018a). Therefore, we concluded that during the processing of PT, oligosaccharide esters were hydrolyzed into secondary glycosides or aglycones *in vivo*, which may be the other reason for the double peak phenomenon.

Combining the changes in *AUC*_0_*_-t_* and *C*_max_ values with the contents of target components in the RPT and various processed PT products, we found that these parameters did not change proportionally. After processing, the contents of A5, A6 and DSS in PT decreased to different degrees, whereas the *AUC*_0_*_-t_* or *C*_max_ values were significantly increased in the LPT group and HPT group, indicating that liquorice boiling and honey stir-baking could enhance the bioavailability of A5, A6 and DSS in PT. Additionally, the content of TFSA in PT decreased during processing, but the two processing methods had no significant effect on the absorption of TFSA *in vivo*. It is worth noting that the content of TMCA in RPT was the lowest, while it was mostly increased in the processed PT products, and the absorption *in vivo* was better in these products than in RPT. The results demonstrated that processing technology could promote the bioavailability of TMCA.

As far as we know, the absorption degree of oral drugs in animals is closely related to their molecular weight, molecular spatial structure and polarity. The molecular weights of A5, A6, DSS, TFSA, TMCA, and IS are 518.47, 548.49, 754.68, 682.23, 238.24 and 138.12 amu, respectively. As shown in Figure 1, the molecular structures of A5 and A6 are similar, and TMCA is the hydrolysate of TFSA. The *t*_1/2_, *AUC*^Δ^_0_*_-t_* and *C*^Δ^_max_ of A5 were larger than those of A6, indicating that the molecular weight might have an important impact on their pharmacokinetic parameters. The absolute bioavailability of TMCA was higher than that of TFSA, which clearly showed that the transformation of the proglucoside to its aglycone form would be conducive to increasing absorption *in vivo*.

In summary, we conclude that the synergistic mechanism of processing PT might be related to some components in liquorice and honey that could promote the absorption of certain oligosaccharide esters and organic acids, such as A5, A6, DSS and TMCA, and the hydrolysis of the oligosaccharide esters into aglycones is beneficial for their absorption by the body. In the future, our team will conduct further verification in combination with pharmacodynamic experiments.

## Conclusions

A fast, accurate, and precise UHPLC-MS/MS method was successfully applied to compare the pharmacokinetic characteristics of A5, A6, DSS, TFSA and TMCA in rats after oral administration of extracts from raw and various processed products of PT, as well as to study the absolute bioavailability differences between the five compounds. This study showed that liquorice boiling and honey stir-baking could enhance the bioavailability of A5, A6 and DSS in PT. It also revealed that the absolute bioavailability of TFSA was significantly lower than that of its aglycone TMCA. This pharmacokinetic study might provide a scientific basis for the clinical application and processing mechanism of processed products of PT.

## Supplementary Material

Supplemental Material

Supplementary_Material.docx
