# Supplementary material for: UHPLC-MS/MS method for pharmacokinetic and bioavailability determination of five bioactive components in raw and various processed products of Polygala tenuifolia in rat plasma
Source: Pharm Biol. 2020 Sep 21;58(1):969–78. doi: 10.1080/13880209.2020.1818790 (PMC7534330; doi:10.1080/13880209.2020.1818790)
Supplement: Supplementary_Material.docx [file IPHB_A_1818790_SM8694.docx]

**Supplementary Material**

**UHPLC-MS/MS method for pharmacokinetic and bioavailability determination of five bioactive components in raw and various processed products of** ***Polygala tenuifolia* in rat plasma**

Xin Zhao^a^*, Baoxin Xu^a^*, Peng Wu^a^, Pan Zhao^a^, Changchuan Guo^b^, Yueli Cui^a^, Yanxue Zhang^a^, Xuelan Zhang^a,c^ and Huifen Li^a^

^a^ School of Chinese Pharmacy, Shandong University of Traditional Chinese Medicine, Jinan, China; ^b^ Shandong Institute for Food and Drug Control, Jinan, China;

^c^ Shandong Provincial Collaborative Innovation Center for Quality Control and Construction of the Whole Industrial Chain of Traditional Chinese Medicine, Jinan, China

CONTACT: Xuelan Zhang, E-mail: zhang8832440@sina.com; Huifen Li, E-mail: lhfen007@126.com. School of Chinese Pharmacy, Shandong University of Traditional Chinese Medicine, Jinan 250355, China.

* These authors contributed equally to this work.

**Content list**

**Table S1.** Linearity and LLOQ data for the five analytes.

**Table S2.** Intra-day and inter-day accuracies and precisions for the five analytes in rat plasma (*n* = 6).

**Table S3.** The extraction recovery and matrix effect for the five analytes in rat plasma (*n* = 6).

**Table S4.** The stability of the five analytes in rat plasma (*n* = 6).

**Figure S1.** HPLC chromatograms of five components in RPT (A), LPT (B) and HPT (C), respectively.

**Table S1.** Linearity and LLOQ data for the five analytes.

| Analytes | Regression equation | Correlation coefficient (*r*) | Linear range (ng/mL) | LLOQ (ng/mL) |
| --- | --- | --- | --- | --- |
| A5 | *y* = 0.000236 *x* + 0.000531 | 0.9964 | 1–2000 | 0.9 |
| A6 | *y* = 0.000272 *x* + 0.000112 | 0.9948 | 1–2000 | 1.0 |
| DSS | *y* = 0.000249 *x* + 0.000311 | 0.9957 | 1–2000 | 0.8 |
| TFSA | *y* = 0.000250 *x* + 0.000336 | 0.9966 | 1–2000 | 0.7 |
| TMCA | *y* = 0.000418 *x* + 0.000449 | 0.9942 | 1–2000 | 0.5 |

**Table S2.** Intra-day and inter-day accuracies and precisions for the five analytes in rat plasma (*n* = 6).

| Analytes | Concentration  (ng/mL) | Intra-day | |  | Inter-day | |
| --- | --- | --- | --- | --- | --- | --- |
|  |  | Accuracy  (RE %) | Precision  (RSD %) |  | Accuracy  (RE %) | Precision  (RSD %) |
| A5 | 2 | 4.2 | 5.4 |  | -6.8 | 5.5 |
|  | 600 | 4.8 | 2.5 |  | 3.2 | 2.7 |
|  | 1600 | -1.1 | 3.6 |  | 0.9 | 0.9 |
| A6 | 2 | 4.2 | 4.6 |  | -7.1 | 7.4 |
|  | 600 | 4.6 | 3.5 |  | 3.1 | 2.1 |
|  | 1600 | -1.9 | 3.5 |  | 1.2 | 2.4 |
| DSS | 2 | 5.3 | 4.3 |  | 4.9 | 7.5 |
|  | 600 | 2.1 | 3.5 |  | 3.2 | 1.8 |
|  | 1600 | 3.9 | 3.5 |  | 4.7 | 2.2 |
| TFSA | 2 | 4.3 | 4.6 |  | 6.8 | 5.5 |
|  | 600 | -3.2 | 3.6 |  | 2.5 | 5.0 |
|  | 1600 | 2.1 | 3.2 |  | -0.9 | 1.1 |
| TMCA | 2 | -4.6 | 6.9 |  | -6.8 | 6.2 |
|  | 600 | 2.5 | 3.1 |  | 2.2 | 3.2 |
|  | 1600 | -0.6 | 1.5 |  | 1.4 | 1.2 |

**Table S3.** The extraction recovery and matrix effect for the five analytes in rat plasma (*n* = 6).

| Analytes | Concentration (ng/mL) | Extraction recovery | |  | Matrix effect | |
| --- | --- | --- | --- | --- | --- | --- |
|  |  | Mean (%) | RSD (%) |  | Mean (%) | RSD (%) |
| A5 | 2 | 85.2 | 3.2 |  | 85.5 | 4.2 |
|  | 600 | 103.4 | 3.8 |  | 91.2 | 4.4 |
|  | 1600 | 98.4 | 3.1 |  | 101.2 | 2.2 |
| A6 | 2 | 92.0 | 4.3 |  | 89.4 | 3.8 |
|  | 600 | 92.4 | 4.3 |  | 88.7 | 2.1 |
|  | 1600 | 96.3 | 3.9 |  | 98.5 | 5.8 |
| DSS | 2 | 85.3 | 5.2 |  | 86.3 | 4.1 |
|  | 600 | 91.2 | 6.2 |  | 95.3 | 4.8 |
|  | 1600 | 98.0 | 3.7 |  | 97.3 | 4.2 |
| TFSA | 2 | 86.1 | 6.2 |  | 88.9 | 5.9 |
|  | 600 | 91.7 | 3.7 |  | 93.8 | 3.6 |
|  | 1600 | 102.5 | 4.3 |  | 103.3 | 2.1 |
| TMCA | 2 | 85.9 | 6.9 |  | 88.6 | 7.2 |
|  | 600 | 96.3 | 3.1 |  | 95.6 | 2.9 |
|  | 1600 | 102.5 | 3.6 |  | 97.3 | 2.1 |

**Table S4.** The stability of the five analytes in rat plasma (*n* = 6).

| Analytes | Concentration  (ng/mL) | Short-term | |  | Freeze-thaw | |  | Auto-sampler | |  | Long-term | |
| --- | --- | --- | --- | --- | --- | --- | --- | --- | --- | --- | --- | --- |
|  |  | Mean (%) | RSD (%) |  | Mean (%) | RSD (%) |  | Mean (%) | RSD (%) |  | Mean (%) | RSD (%) |
| A5 | 2 | 94.4 | 3.2 |  | 92.1 | 5.6 |  | 89.4 | 4.9 |  | 88.6 | 3.2 |
|  | 600 | 95.2 | 4.5 |  | 93.5 | 5.7 |  | 94.2 | 4.4 |  | 97.9 | 2.7 |
|  | 1600 | 98.3 | 2.1 |  | 101.6 | 3.6 |  | 98.7 | 1.9 |  | 96.6 | 2.7 |
| A6 | 2 | 91.5 | 3.6 |  | 92.4 | 4.2 |  | 87.5 | 5.7 |  | 93.5 | 4.6 |
|  | 600 | 94.7 | 5.1 |  | 91.5 | 3.6 |  | 92.1 | 5.1 |  | 96.3 | 1.1 |
|  | 1600 | 97.4 | 3.2 |  | 98.3 | 3.5 |  | 97.3 | 5.2 |  | 96.5 | 3.7 |
| DSS | 2 | 95.3 | 5.8 |  | 89.3 | 6.7 |  | 92.1 | 5.6 |  | 95.3 | 3.1 |
|  | 600 | 98.5 | 5.1 |  | 97.3 | 4.9 |  | 97.7 | 4.6 |  | 97.3 | 2.0 |
|  | 1600 | 98.1 | 3.7 |  | 99.7 | 2.0 |  | 98.6 | 3.6 |  | 93.2 | 4.2 |
| TFSA | 2 | 97.6 | 3.1 |  | 96.5 | 5.9 |  | 96.6 | 5.3 |  | 89.9 | 3.6 |
|  | 600 | 97.9 | 3.6 |  | 96.3 | 4.7 |  | 98.2 | 3.7 |  | 96.3 | 2.2 |
|  | 1600 | 98.5 | 4.2 |  | 97.4 | 2.6 |  | 96.3 | 5.9 |  | 96.2 | 2.8 |
| TMCA | 2 | 96.2 | 4.9 |  | 95.3 | 3.1 |  | 96.3 | 3.7 |  | 98.3 | 2.6 |
|  | 600 | 98.3 | 2.1 |  | 97.3 | 2.9 |  | 99.8 | 4.2 |  | 95.5 | 2.8 |
|  | 1600 | 102.5 | 3.2 |  | 98.8 | 3.1 |  | 99.5 | 4.2 |  | 95.8 | 3.3 |
